# Periostin Responds to Mechanical Stress and Tension by Activating the MTOR Signaling Pathway

**DOI:** 10.1371/journal.pone.0083580

**Published:** 2013-12-13

**Authors:** Luciana K. Rosselli-Murai, Luciana O. Almeida, Chiara Zagni, Pablo Galindo-Moreno, Miguel Padial-Molina, Sarah L. Volk, Marcelo J. Murai, Hector F. Rios, Cristiane H. Squarize, Rogerio M. Castilho

**Affiliations:** 1 Laboratory of Epithelial Biology, Department of Periodontics and Oral Medicine, School of Dentistry, University of Michigan, Ann Arbor, Michigan, United States of America; 2 Department of Oral Surgery and Implant Dentistry, School of Dentistry, University of Granada, Granada, Spain; 3 The Division of Hematology and Oncology, University of Michigan Comprehensive Cancer Center, Ann Arbor, Michigan, United States of America; 4 Department of Periodontics and Oral Medicine, School of Dentistry, University of Michigan, Ann Arbor, Michigan, United States of America; University of Birmingham, United Kingdom

## Abstract

Current knowledge about Periostin biology has expanded from its recognized functions in embryogenesis and bone metabolism to its roles in tissue repair and remodeling and its clinical implications in cancer. Emerging evidence suggests that Periostin plays a critical role in the mechanism of wound healing; however, the paracrine effect of Periostin in epithelial cell biology is still poorly understood. We found that epithelial cells are capable of producing endogenous Periostin that, unlike mesenchymal cell, cannot be secreted. Epithelial cells responded to Periostin paracrine stimuli by enhancing cellular migration and proliferation and by activating the mTOR signaling pathway. Interestingly, biomechanical stimulation of epithelial cells, which simulates tension forces that occur during initial steps of tissue healing, induced Periostin production and mTOR activation. The molecular association of Periostin and mTOR signaling was further dissected by administering rapamycin, a selective pharmacological inhibitor of mTOR, and by disruption of Raptor and Rictor scaffold proteins implicated in the regulation of mTORC1 and mTORC2 complex assembly. Both strategies resulted in ablation of Periostin-induced mitogenic and migratory activity. These results indicate that Periostin-induced epithelial migration and proliferation requires mTOR signaling. Collectively, our findings identify Periostin as a mechanical stress responsive molecule that is primarily secreted by fibroblasts during wound healing and expressed endogenously in epithelial cells resulting in the control of cellular physiology through a mechanism mediated by the mTOR signaling cascade.

## Introduction

The human body is protected from biological, physical, and chemical insults by a physical barrier comprised of epithelial and stromal cells that constitute the skin. The skin is primarily responsible for preventing water loss by maintaining tissue integrity and by responding to injuries in a controlled and time-dependent manner [1-4]. Following injury, compromised structures undergo a prolonged period of tissue remodeling that culminates in the recovery of skin protective functions.

 Recently, new molecules, including Periostin, have been associated with the wound healing process. Periostin is found in normal skin, during tissue repair, and in pathological conditions, such as cancer [5-9]. Notably, Periostin (also called OSF-2 and encoded by the *POSTN* gene) is found in tissues involved in mechanical stress conditions, such as periodontal ligaments, periosteum [10] and cardiac valves [11], where it is secreted into the extracellular matrix following acute injury to the heart [12], skin [6,13] and others tissues [14,15]. Furthermore, recent studies have shown increased Periostin expression and deposition in fibrotic conditions, including keloid and hyperplastic scarring of the skin [13]. 

 New insights into the role of Periostin in cutaneous wounds came from analyzing its effect in mouse dermal fibroblasts and in myofibroblast differentiation [7,8,16,17]. However, the effect of Periostin signaling on epithelial response and other molecular circuitry is poorly understood. We show that Periostin is primarily secreted from fibroblasts and confers a paracrine effect in human keratinocyte proliferation and migration. The mechanisms underlying Periostin-induced migration are associated with activation of mTOR circuitry, as evidenced by phosphorylation of AKT at threonine 308 and serine 473 and the mTOR downstream molecule S6. Interestingly, we also found that upregulation of Periostin following mechanical stress was accompanied by mTOR overexpression; and their combined effects orchestrated the migratory response of epithelial cells. Indeed, pharmacological inhibition of mTOR by rapamycin and by siRNA targeting Raptor and Rictor, which disrupted mTORC1 and mTORC2 complexes respectively, resulted in reduced migration and proliferation of epithelial cells. Collectively, these findings indicate that Periostin responds to mechanical stress during wound healing to induce proliferation and migration by a mechanism that requires activation of the PI3K/mTOR signaling pathway.

## Material and Methods

### Ethics Statement

This animal study was performed according to the University of Michigan Committee on Use and Care of Animals (UCUCA) approved protocol (protocol # 10428) and in compliance with the Guide for the Care and Use of Laboratory Animals. Animals were housed in 12-hrs light/dark cycles and received standard rodent chow and water ad libitum in compliance with AAALAC guidelines. Investigators and animal care staff observed the animals daily. Mice showing discomfort, wasting, hunching, or other signs indicative of distress were treated appropriately to alleviate discomfort or were euthanized.

### Experimental Mice and *In Vivo* Wound Healing Assay

The *in vivo* wound healing assays were performed in the shaved skin on the dorsal surface. Fifteen millimeters full-thickness incisional epidermal wounds were made in the mid-dorsal area. At day four after skin wounding, freshly prepared 5-bromo-2’-deoxyuridine (BrDU) was injected intraperitoneally (I.P.) at a concentration of 100 μg/g body weight 2 hours before sacrificing the animals. Wound fields were excised, fixed in 10% aqueous buffered zinc formalin, paraffin embedded, and sectioned.

### Histology and Immunohistochemistry

Hematoxylin and eosin (H&E) staining was performed on sections from formalin-fixed and paraffin-embedded tissue according to standard procedures. Immunohistochemistry assays were performed on serial sections after antigen retrieval using primary antibodies against BrDU (Axyll-Accurate Chemical & Scientific Corporation, Westbury, NY), Cytokeratin 6 (K6) (169P, Covance) and Periostin (RD181045050, Biovendor) and biotinylated secondary antibodies (BA-1000, Vector Laboratories). Sections were washed, incubated with avidin-biotin complex (ABC kit, Vector Laboratories) and developed using the DAB substrate kit (Sigma-Aldrich). Slides were analyzed and photographed using a Nikon Eclipse 80i Microscope (Nikon, Melville, NY).

### Cell cultures

The normal oral keratinocyte spontaneously immortalized (NOK-SI) cell line [18] was cultured in Dulbecco’s Modified Eagle Medium (DMEM) supplemented with 10% fetal bovine serum (FBS), 100 U/ml penicillin G, 100 µg/ml streptomycin, and 250 ng/ml amphotericin B at 37°C in a humid 5% CO_2_ atmosphere. Primary human PDL fibroblasts (hPDL) [19] were used between passages 4 and 7 and maintained in DMEM with sodium pyruvate and supplemented with 10% FBS, 100 U/ml penicillin G, 100 µg/ml streptomycin, 250 ng/ml amphotericin B and 2 mM glutamine at 37°C in a humid 5% CO_2_ atmosphere.

### Conditioned media from NOK-SI and hPDL cells

Subconfluent monolayers of NOK-SI and hPDL cells were cultured in DMEM and supplemented with 10% FBS. After 48 hours, media were collected and concentrated 6x by centrifugation using Ultracel-30 Centrifugal membrane filters (30 kDa) (Millipore, Billerica, MA, USA) following manufacturer instructions. Concentrated conditioned media were mixed with SDS electrophoresis loading buffer for western blot analysis. 

### In vitro scratch wound assay, immunofluorescence, and proliferation

NOK-SI cells were plated at high confluence followed by a mechanical scratch. Each well was treated with 50 ng/ml Periostin and/or 50 nM rapamycin. Control cells were treated with vehicle, and EGF served as a positive control. Quantitative analyses of the open areas were performed using Axionvision software (Carl Zeiss, Germany) at indicated time points. F-actin staining was performed on glass coverslips 12 hours after scratching. Cells were washed with cold PBS, fixed with fresh 4% paraformaldehyde (PFA) and permeabilized with 0.1% Triton for 5 min. Cells were incubated with 100 nM phalloidin-rhodamine for 30 min (Cytoskeleton, Denver, USA) and then stained with Hoechst 33342. Cells were also stained with anti-Periostin (Biovendor R&D, Candler, USA) and anti-pS6 (Dako, Carpinteria, USA) antibodies and then incubated with FITC or TRITC secondary antibodies. Images were taken with a QImaging ExiAqua monochrome digital camera attached to a Nikon Eclipse 80i Microscope and QCapturePro software (Nikon, Melville, NY). NOK-SI cell proliferation was assessed following treatment with the rapid colorimetric MTT assay kit (Trevigen Inc.) according to manufacturer's instruction.

### Western blot analysis

Cells were harvested after indicated times, treated with RIPA buffer and sonicated briefly. Protein lysates (30μg) were separated by 10% SDS-PAGE gel and transferred to a polyvinyl difluoride membrane (PVDF-Immobilon, Millipore). Membranes were blocked in 0.1 M Tris (pH 7.5), 0.9% NaCl and 0.05% Tween-20 (TBS-T) containing 5% nonfat dry milk and probed with anti-Periostin (Biovendor), pS6 (Dako), pAKT^T308^ (Abcam) and pAKT^S473^ (Cell Signaling) antibodies. GAPDH (Calbiochem) was used as a loading control. Membranes were incubated with appropriate secondary antibodies conjugated with horseradish peroxidase, and bands were detected using ECL SuperSignal West Pico Substrate (Pierce Biotechnology).

### Raptor and Rictor Knockdown

Knockdown of Raptor and Rictor was performed in NOK-SI cell lines as previously described [18,20,21]. Briefly, cells were seeded in 24-well plates and transfected with 15 nM double-stranded RNA oligonucleotides directed against human Raptor (forward: 5’- GGA CAA CGG CCA CAA GUAdTdT-3’ and reverse: 5’- UAC UUG UGG CCG UUG UCCdTdT-3’) and 5 nM double-stranded RNA oligonucleotides against Rictor (forward: 5’- CCU AAU GAA UAU GGC UGC AUC CUU UdTdT-3’ and reverse: 5’- AAA GGA UGC AGC CAU AUU CAU UAG GdTdT-3’) (Invitrogen). Optimal concentrations and time points were determined by dilution curves of siRNA for each target and immunoblot analysis. The sequences of the control negative siRNA (Invitrogen) oligonucleotides were as follows: 5′-UUC UCC GAA CGU GUC ACG UdTdT-3′ and 5′-ACG UGA CAC GUU CGG AGA AdTdT-3′ [22].

### Biomechanical stimulation

NOK-SI and hPDL cells were plated in flexible bottom 6-well plates (BioFlex™ Culture Plates, Flexcell, USA) coated with Collagen I at a density of 250,000 cells/well. At 24h post-seeding, cells were subjected to biomechanical stimulation (i.e., 14% stretching at 6 cycles/min) using the Flexcell FX-5000 cell tension system (Flexcell, USA). Non-stretched cells served as controls. At indicated time points, cells were lysed and subjected to western blot analysis.

### Statistical analysis

 Statistical analyses were performed using GraphPad Prism 5 (GraphPad Software, San Diego, CA). Statistical analyses of cellular proliferation and migration assays were performed using Student’s t test and ANOVA-one-way analysis followed by Newman-Keuls or Bonferroni multiple comparison test. Asterisks denote statistical significance (*p<0.05; **p<0.01; ***p<0.001; and NS p>0.05). 

## Results

### Periostin is expressed in epithelial cells undergoing wound-healing associated stress

**Figure 1:**
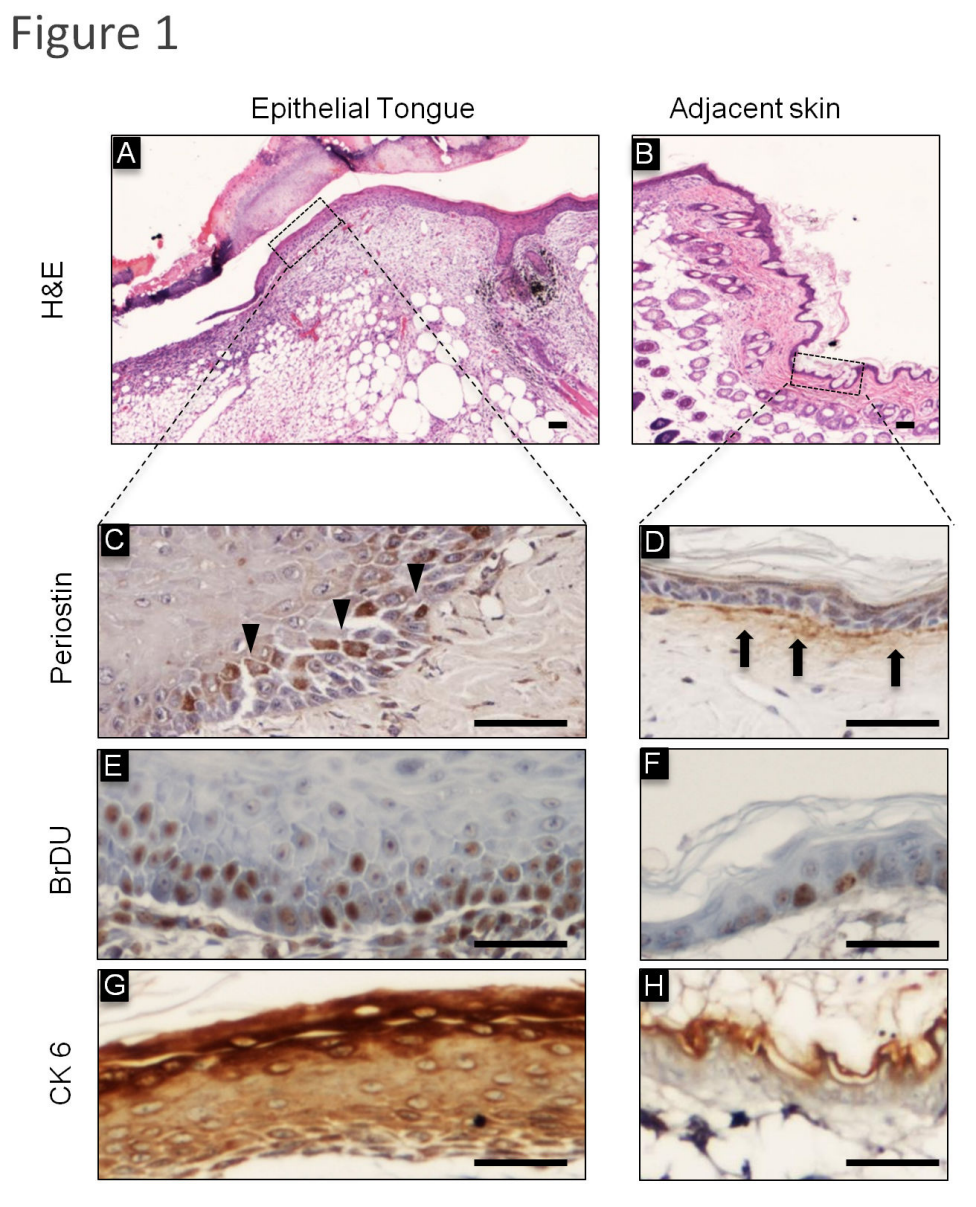
Expression of Periostin and CK6 during cutaneous wound healing. **H**&**E**: Representative histological sections of cutaneous incisional wounds. (**A**) Morphology of the wounded site shows a thin edge of epithelial cells migrating across the wound bed, termed the epithelial tongue and (**B**) intact and normal skin adjacent to the wounded site were stained with Hematoxylin and Eosin (H&E). (**C**) Epithelial cells at the epithelial tongue express intracellular Periostin. (**D**) In normal adjacent skin, Periostin is in the connective tissue at the basal lamina, which is juxtaposed to the epithelial basal layer. (**E**) Note that basal and parabasal layers of the epithelial tongue have a large number of proliferating BrDU positive cells. (**F**) As expected, the epithelial basal layer of adjacent skin has few proliferating cells. (**G**) Upregulation of the epithelia stress/tension marker CK6 is depicted in the epithelial tongue compared to normal adjacent skin observed in (**H**). Scale bars represent 50 μm.

Following skin injury, epithelial cells undergo an intricate process of cellular proliferation and differentiation. During wound repair, keratinocytes exhibit increased proliferation, which results in thickening of the epidermis and formation of an epithelial edge, characterized by enhanced motility [18,23,24]. Epithelial cells on the wound margins start to proliferate, resulting in thickening of the epidermis, at days 2-3 post-wounding and cell migration towards the wound bed is initiated. As seen in Figure 1A, the migrating keratinocytes form a fine wedge shape known as the epithelial tongue, which is constituted by one or two cell layers, which establish the leading wound edge by moving over the granulation tissue and under the fibrin clot [1,25,26]. Interestingly, Periostin was found differentially expressed in the actively migrating epithelial tongue and the adjacent epidermis observed under homeostasis. Notably, Periostin was expressed under the epithelium, specifically in the basement membrane of the skin, but epithelial cells lacked Periostin expression (Figure 1B and 1D). Interestingly, epithelial cells undergoing active migration (Figure 1A) showed accumulation of intracellular Periostin that was localized at the cytoplasm (Figure 1C). Increased levels of Periostin in epithelial cells were associated with elevated proliferation (Figure 1E) compared to adjacent epithelial cells in homeostasis (Figure 1F), as detected by short pulse BrDU incorporation. Indeed, BrDU accumulation was evident in the basal and parabasal layers of the epidermis, similar to what is observed with Periostin staining (Figure 1C and 1E). 

Periostin expression has been reported in diverse organs and conditions, including the heart, the periosteum and the periodontal ligament [27,28]. Interestingly, organs that express Periostin are associated with mechanical stress, as demonstrated by the normal physiology of heart valves and periodontal ligaments undergoing mechanical tension [10,11]. In epithelial cells, mechanical stress and tension is detected by expression of CK6 [18]. During wound healing, epithelial cells responsible for closing the wound are stretched along the wound borders and express CK6 throughout the epithelial tongue layers (Figure 1G); however, CK6 is not expressed in normal epithelium under homeostasis (Figure 1H), indicating that mechanical stress and tension is involved in wound closure.

### Fibroblast-Secreted Periostin Induces Paracrine Activation of Epithelial Migration and Proliferation

**Figure 2:**
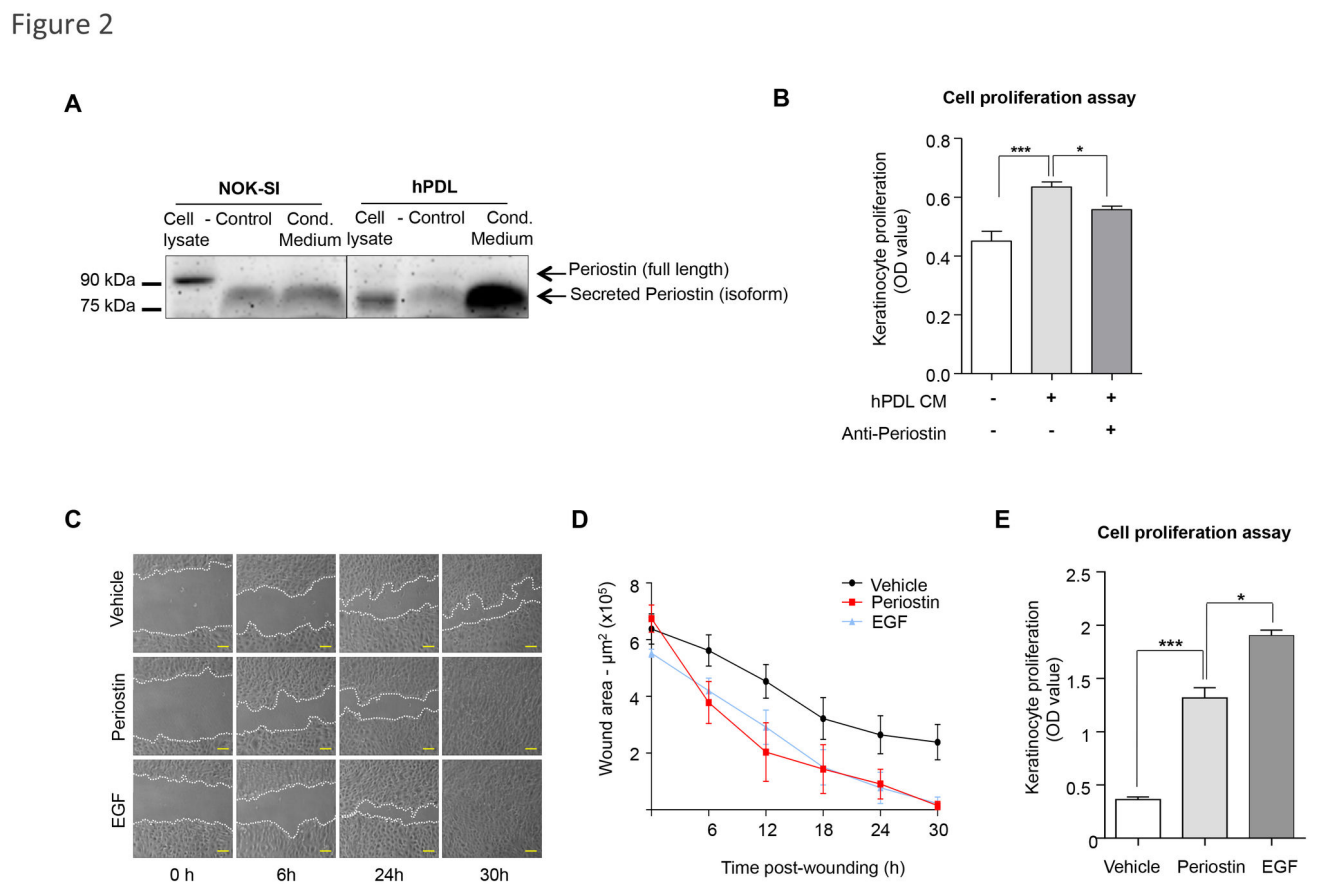
Periostin-driven migration and proliferation. (**A**) Total cell lysates and conditioned medium (cond. medium) from NOK-SI and hPDL cells were blotted for Periostin. New cell culture medium supplemented with 10% FBS was used as a negative control (control). Intracellular Periostin is detected in epithelial cell lysate. However, conditioned medium from NOK-SI shows that keratinocytes did not secrete Periostin, as the same band was observed in the negative control media. hPDL cells have low levels of the intracellular Periostin isoform as observed in the cell lysate. Increased levels of secreted Periostin were found in the conditioned medium from hPDL. (**B**) hPDL conditioned medium induces keratinocyte proliferation compared to vehicle alone (***p<0.001), which is reduced upon administration of anti-Periostin antibody (*p<0.05). (**C**) Representative pictures of NOK-SI migration following treatment with recombinant Periostin (50 ng/ml), EGF (100 ng/ml) as the positive control, or vehicle. Scale bars represent 50 μm. (**D**) Graphic represents the quantification of the wound areas at indicated times (n=4; mean ± S.E.M). (**E**) Periostin enhances proliferation of keratinocytes compared to vehicle treated cells (***p<0.001). EGF treatment was used as positive control (*p<0.05) (n=6; mean ± S.E.M).

A few regulatory mechanisms associated with Periostin expression in the epidermis and dermis have been elucidated [8,29], but the physiological impact of Periostin expression and secretion by epithelial and stromal cells in skin biology is largely unknown. We first examined Periostin production and secretion by epithelial and mesenchymal cells. Using a human keratinocyte cell line (NOK-SI) [18] and a primary culture of human fibroblasts (hPDL) [30], we found that both cell types produced Periostin (Figure 2A). Full length Periostin is a 93 kDa protein, and additional human Periostin isoforms range from 83 kDa to 87 kDa (from UniProtKB/Swiss-Prot database - Q15063). When secreted, Periostin isoforms become part of the extracellular matrix. NOK-SI produced full-length Periostin, and hPDL produced and secreted a Periostin isoform (Figure 2A). To examine Periostin secretion from NOK-SI and hPDL cells, we analyzed their conditioned medium. We found that fibroblast cells produced and secreted large amounts of the Periostin isoform (Figure 2A). NOK-SI cells did not secrete Periostin, as evidenced by similar Periostin detection in the negative control (DMEM supplemented with 10%FBS) and NOK-SI conditioned medium alone, suggesting that Periostin expression in NOK-SI is a byproduct of serum-supplemented culture media (Figure 2A). Next, in order to understand the physiological effect of fibroblast-secreted Periostin on epithelial cell biology, we treated NOK-SI cells with conditioned medium from hPDLs. Interestingly, conditioned medium enhanced proliferation of epithelial cells compared to vehicle treated cells (Figure 2B, ***p<0.001). To confirm that Periostin was acting as a paracrine mitogenic effector originated from fibroblast condition medium, competitive assay using anti-Periostin antibody revealed a reduction in proliferation (Figure 2B, **p<0.01). Additionally, much like the secreted isoform, recombinant Periostin enhanced epithelial migration (Figure 2C and 2D). Indeed, Periostin induced robust cellular migration that was similar to the epidermal growth factor (EGF) positive control. In addition to migration, full-length Periostin also enhanced the proliferation of keratinocytes (Figure 2E, ***p<0.001).

### Periostin induces stress fiber formation and activation of the AKT/mTOR signaling pathway

**Figure 3:**
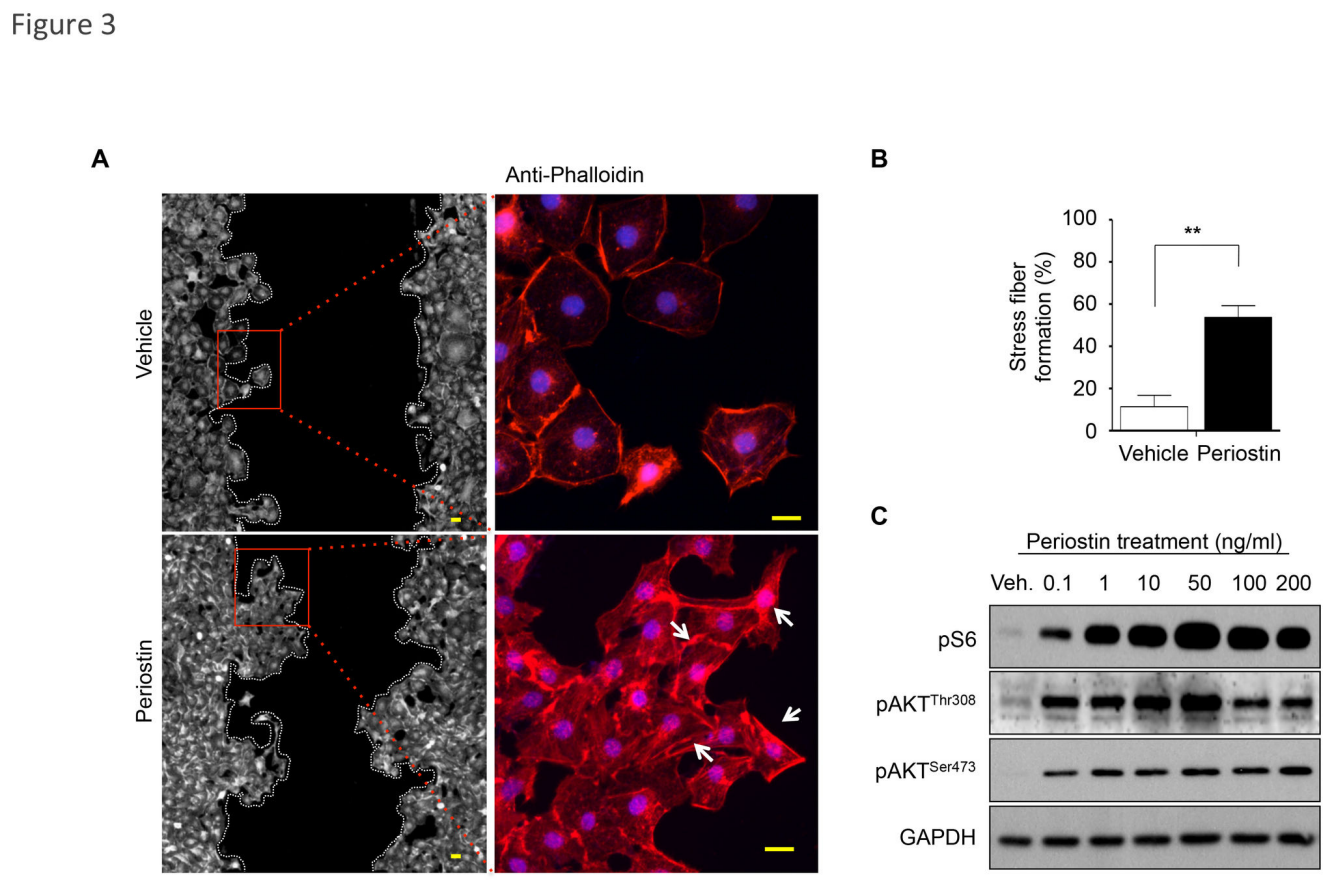
F-actin polarization and PI3K/mTOR signaling activation by Periostin-induced epithelial cell migration. (**A**) Phalloidin detection shows cells with polarized F-actin (white arrow) following treatment with recombinant Periostin compared to vehicle control. Scale bars represent 10 μm. (**B**) Graphic represents percentage of cells with stress fiber formation (polarized F-actin) after periostin or vehicle stimuli. Results were determined by measuring fields using independent triplicates (**p<0.01) (**C**) Activation of PI3K and mTOR signaling is triggered by Periostin treatment in a dose-dependent manner, as detected by phosphorylated AKT at Threonine 308 (pAKT^Thr308^) and Serine 473 (pAKT^Ser473^) and phosphorylated S6 (pS6). Note that 50 ng/ml of Periostin is the optimal concentration for PI3K activation. GAPDH was used as a loading control.

We next examined the molecular signaling involved in Periostin-induced epithelial migration and proliferation. Using an *in vitro* wound scratch assay, we found that Periostin induced the polarization pattern of F-actin cytoskeleton filaments. As revealed by phalloidin staining, F-actin filaments were predominantly localized at the cellular periphery in vehicle-treated NOK-SI cells, but Periostin-treated cells showed strong actin polymerization and morphological changes, such as a spindle-like shape (Figure 3A and 3B), similar to the lamellipodial protrusions found in highly motile cells [31,32]. Emerging evidence suggests that mTORC1 pathway coordinates changes in cell morphology [33]. Here, we found that Periostin induced dose-dependent activation of AKT/mTOR signaling, evidenced by phosphorylation of pS6 and AKT at threonine 308 (pAKT**^Thr308^**). Additionally, upon Periostin stimuli, AKT was also activated at serine 473 (pAKT**^Ser473^**) compared to the vehicle (control), but its level remained constant thereafter (Figure 3C). Because mTORC2 complex activation results in phosphorylation of AKT at Ser473 [34,35] and S6 phosphorylation is mediated by mTORC1, our results suggest that Periostin signaling is mostly mediated through mTORC1. Periostin activated AKT/mTOR signaling at very low concentrations (i.e., 0.1 ng/ml) relative to vehicle (Figure 3C). Interestingly, Periostin-induced activation of AKT/mTOR peaked at 50 ng/ml. Higher concentrations of Periostin (100 and 200 ng/ml) caused a reduction in AKT/mTOR expression. These results suggest that the effect of Periostin on AKT/mTOR activation is concentration dependent, and 50 ng/ml is the optimal dose for inducing AKT and mTOR phosphorylation.

### Biomechanical stimulation of epithelial cells triggers endogenous Periostin expression and mTOR signaling

**Figure 4:**
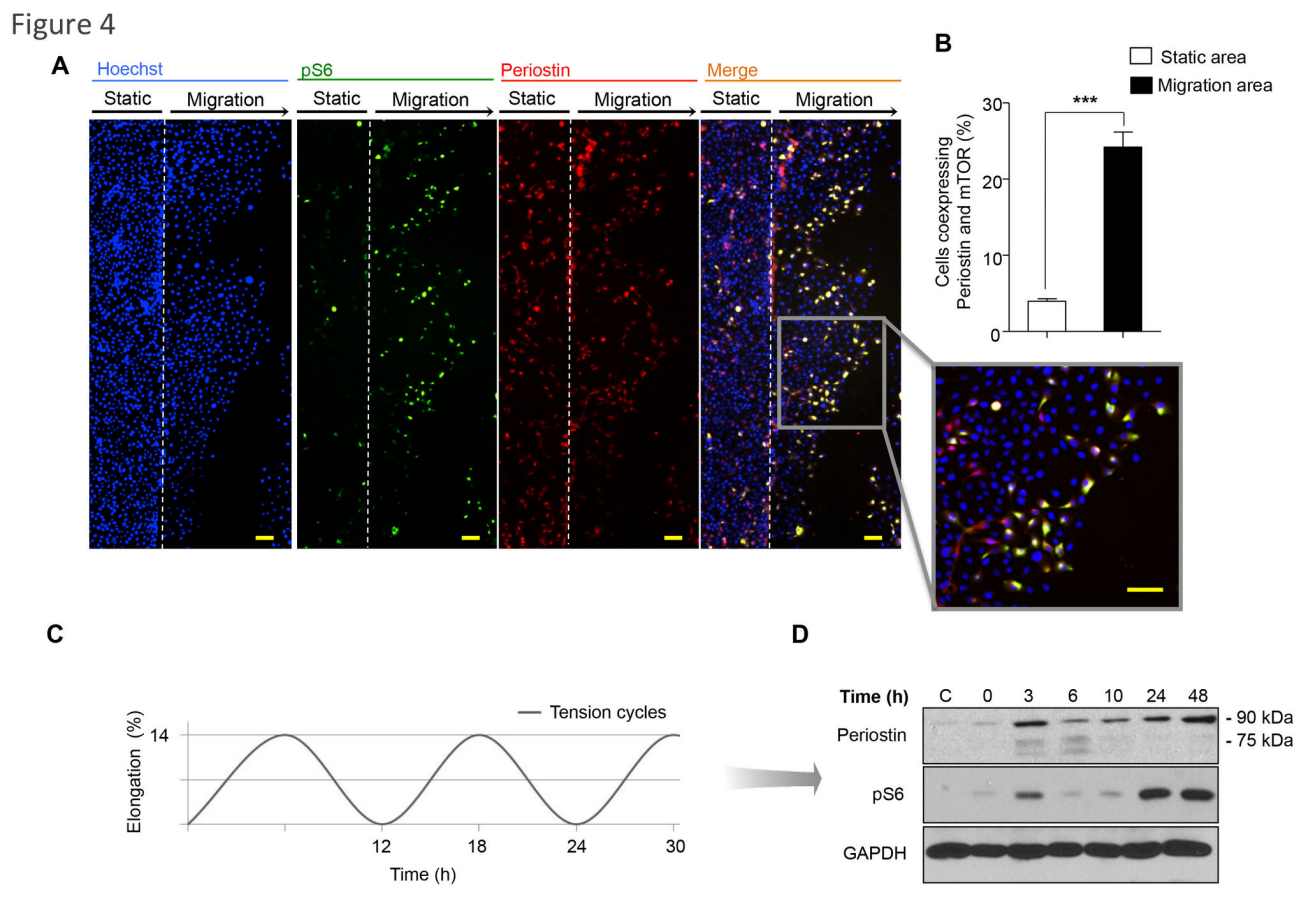
Co-expression of Periostin and mTOR during cellular migration and mechanical stress induced by tension. (**A**) A representative wound healing scratch assay shows keratinocytes stained for Periostin (TRITC-red), pS6 (FITC-green) and DNA (Hoechst-blue). Note colocalization of Periostin and pS6 staining (on merge and insert) in the migratory area. Scale bars represent 50 μm. (**B**) Quantification of positive cells co-expressing Periostin and pS6 are depicted. Note increased number of positive cells co-expressing Periostin and pS6. Most of these cells are in the migratory area (***p<0.001). (**C**) NOK-SI cells were subjected to biomechanical stimulation (load of 14% stretching at 6 cycles/min) at the indicated time points. (**D**) Western blot analysis for Periostin and pS6 expression in NOK-SI subjected to load assay. Non-stimulated cells (no load force) served as a control, and GAPDH was used as protein loading control.

Tension-associated cellular polarization appears to play a major role in a wide range of migratory cells. Cellular tension can vary from protrusion to contraction and with tension-bearing structures associated with the membrane or cytoskeleton. However, the molecular mechanisms underlying the translation of mechanical stress into migratory signaling are largely unknown in epithelial biology. Periostin is upregulated in diverse tissues exposed to continuous tension, including the periosteum, where its mechanical properties are key to maintaining bone strength [28,36]. Periostin is also expressed in various soft tissues that are under continuous mechanical stress, including the heart and heart valves [11,27], tendons [37], cornea [38] and periodontal ligament [39,40]. Emerging evidence indicates that mechanical stress plays a key role in Periostin expression and signaling. We wanted to determine the effects of mechanical tension in epithelial cell biology by examining Periostin and mTOR expression. In particular, collective cell guidance of epithelial cells in culture after wound scratch results in a dynamic interaction of intercellular stress forces [41]. We found that migratory epithelial cells co-expressed Periostin and the active pS6 mTOR signaling marker at the leading edge of the migratory front (Figure 4A and insert), corresponding to a five-fold increase in Periostin and pS6 colocalization compared to static cells (***p<0.001)(Figure 4B). To further characterize the impact of mechanical stress on Periostin expression and activation of mTOR, we subjected NOK-SI cells to a computer-controlled biomechanical stimulation of stress and tension. NOK-SI cells were exposed to various magnitudes of tensile strain for 0, 3, 6, 10, 24 and 48 hours (Figure 4C) and analyzed for Periostin and pS6 protein expression. Western blot analysis revealed increased Periostin after 3 hours of tension cycles, with greater expression at 24 and 48 hours. Notably, mTOR activation followed exactly the same pattern as Periostin expression (Figure 4D), indicating a strong relationship among Periostin, AKT/mTOR activation and mechanical tension. 

### Periostin-induced epithelial migration is dependent on mTORC1 signaling

**Figure 5:**
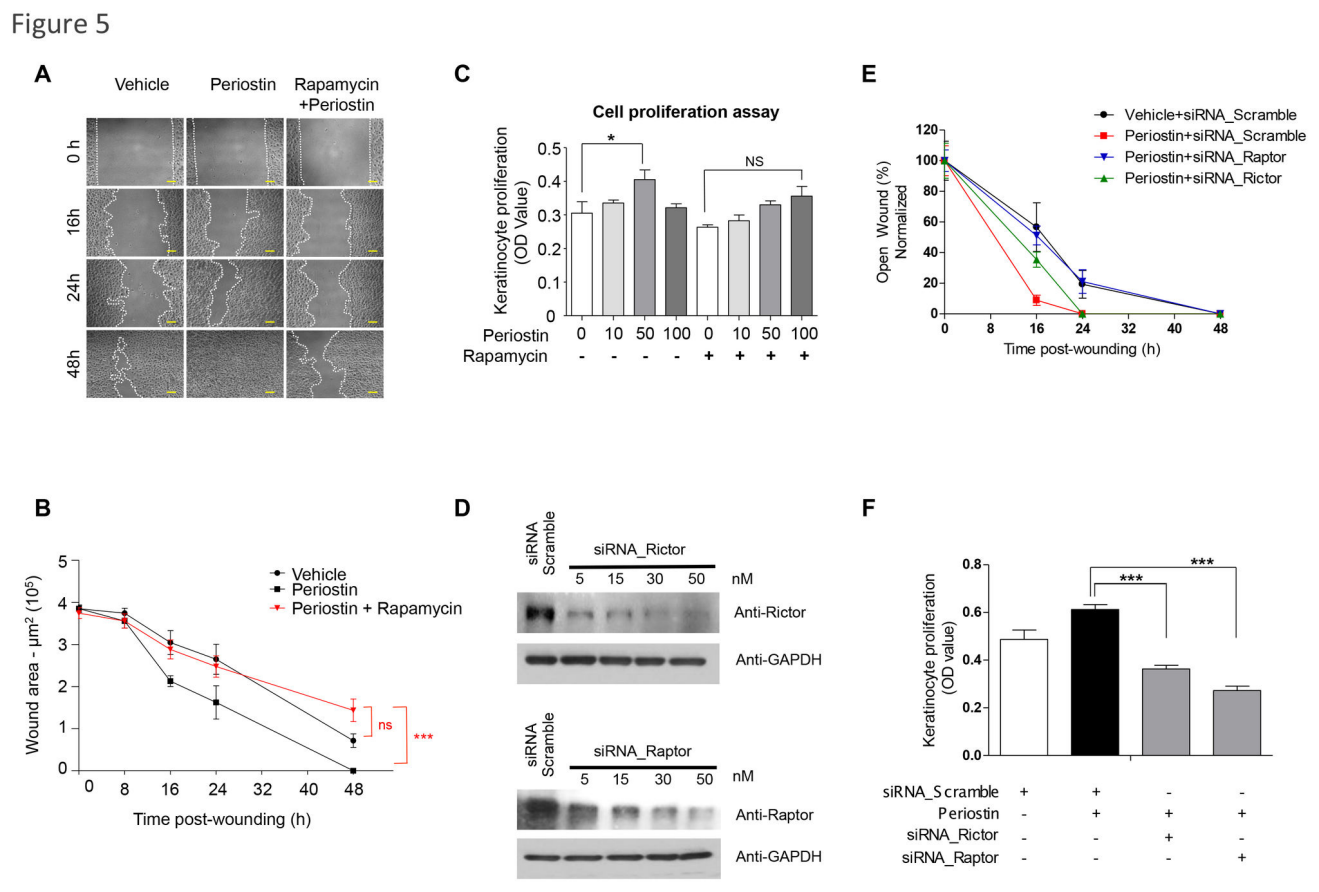
Periostin-driven cellular migration requires mTOR signaling. (**A**) Representative pictures of the NOK-SI cell scratch assay following treatment with vehicle, recombinant Periostin (50 ng/ml), and rapamycin (50 nM). Scale bars represent 50 μm. (**B**) Quantitative analysis of open-wounded area over time (n=4; mean ± S.E.M.). Note that rapamycin abrogates the Periostin migratory activity of epithelial cells (***p<0.001). (**C**) Proliferation assay using keratinocytes treated with rapamycin and/or Periostin. Note that Periostin alone induced significant cellular proliferation at 50 ng/ml (*p<0.05). Treatment with rapamycin blocked periostin-induced cell proliferation (ns p>0.05). (**D**) Representative immunoblot depicting knockdown of Raptor and Rictor after siRNA treatment. Scramble siRNA oligonucleotides sequences were used as controls. GAPDH was used as loading controls. (**E**) Graphic shows the quantitative analyses of open-wounded areas using NOK-SI cells over time (n=4; mean ± S.E.M.). Note that siRNA targeting Raptor abrogates Periostin-induced cellular migratory resulting on complete wound closure by 48 hours (**p<0.05). siRNA targeting Rictor did not change the Periostin induced accelerated cellular migration resulting on wound closure by 24 hours (ns p>0.05). (**F**) Proliferation assay using NOK-SI cells treated with siRNA for Raptor, Rictor, or siRNA scramble, and/or Periostin. Note that Periostin induced significant cellular proliferation (*p<0.05). Treatment with siRNA for Raptor or Rictor resulted in disruption of Periostin induced cellular proliferation (***p<0.001).

 We previously demonstrated the involvement of mTOR signaling in accelerated epithelial migration using genetically defined mouse models and showed that *in vivo* inhibition of mTOR using rapamycin directly impacts cutaneous healing [23]. However, the molecular circuitry involved in mTOR-induced cellular migration is still poorly understood. These observations prompted us to explore the requirements for mTOR signaling during Periostin driven accelerated epithelial migration. For this, we took advantage of the ability of rapamycin to selectively inhibit mTOR signaling [42] and performed a scratch assay using NOK-SI cells. NOK-SI cells treated with vehicle showed near closure of the open scratch within 48 hours, but the accelerated epithelial migration stimulated by Periostin became evident within the first 16 hours of cellular migration and completely closed the scratch wound within 48 hours. Furthermore, administration of rapamycin abrogated Periostin-induced cell migration, resulting in migration rates similar to vehicle control (Figure 5A and 5B ***p<0.001). These results suggest that mTOR signaling is required for Periostin-driven cellular migration. Additionally, we found that cell proliferation increased in a dose-dependent manner following Periostin treatment at concentrations from 10 to 50 ng/ml (*p<0.05); inhibition was observed with 100 ng/ml of Periostin (Figure 5C). Notably, such inhibition correlates with the ability of Periostin to induce activation of mTOR signaling at an optimal concentration of 50 ng/ml, which is followed by a reduction in AKT (pAKT**^Thr308^**) and pS6 activation (Figure 3C). Next, we found that although cells continue to display Periostin-induced proliferation (Figure 5C; * p<0.05), this growth was not statistically significant after rapamycin administration (Figure 5C; ns p>0.05). These findings along our previous results suggest that Periostin may signal through the mTORC complexes to induce migration and proliferation. To further dissect this molecular mechanism, we interfered with mTORC1 and mTORC2 complexes by disrupting its correspondent scaffold proteins, Raptor [43,44] and Rictor [45,46] (Figure 5D). We found that by interfering with Raptor, NOK-SI cells shown reduced migration upon administration of Periostin requiring 48 hours to close the wound (**p<0.05). Interference of Rictor only marginally impaired cellular migration at 16 hours of wound (ns p>0.05) and did not interfere with Periostin–driven accelerated wound closure, which closed at 24hrs after wounding along with the positive controls receiving Periostin and scrambled siRNA (Figure 5E). Notably, interference of Raptor and Rictor resulted in significant reduction of proliferation (**p<0.05)(Figure 5F). These findings suggest that Periostin-driven epithelial migration is mainly mediated by mTORC1 signaling, while cellular proliferation involves the two complexes, mTORC1 and mTORC2 (Figure 5F).

**Figure 6:**
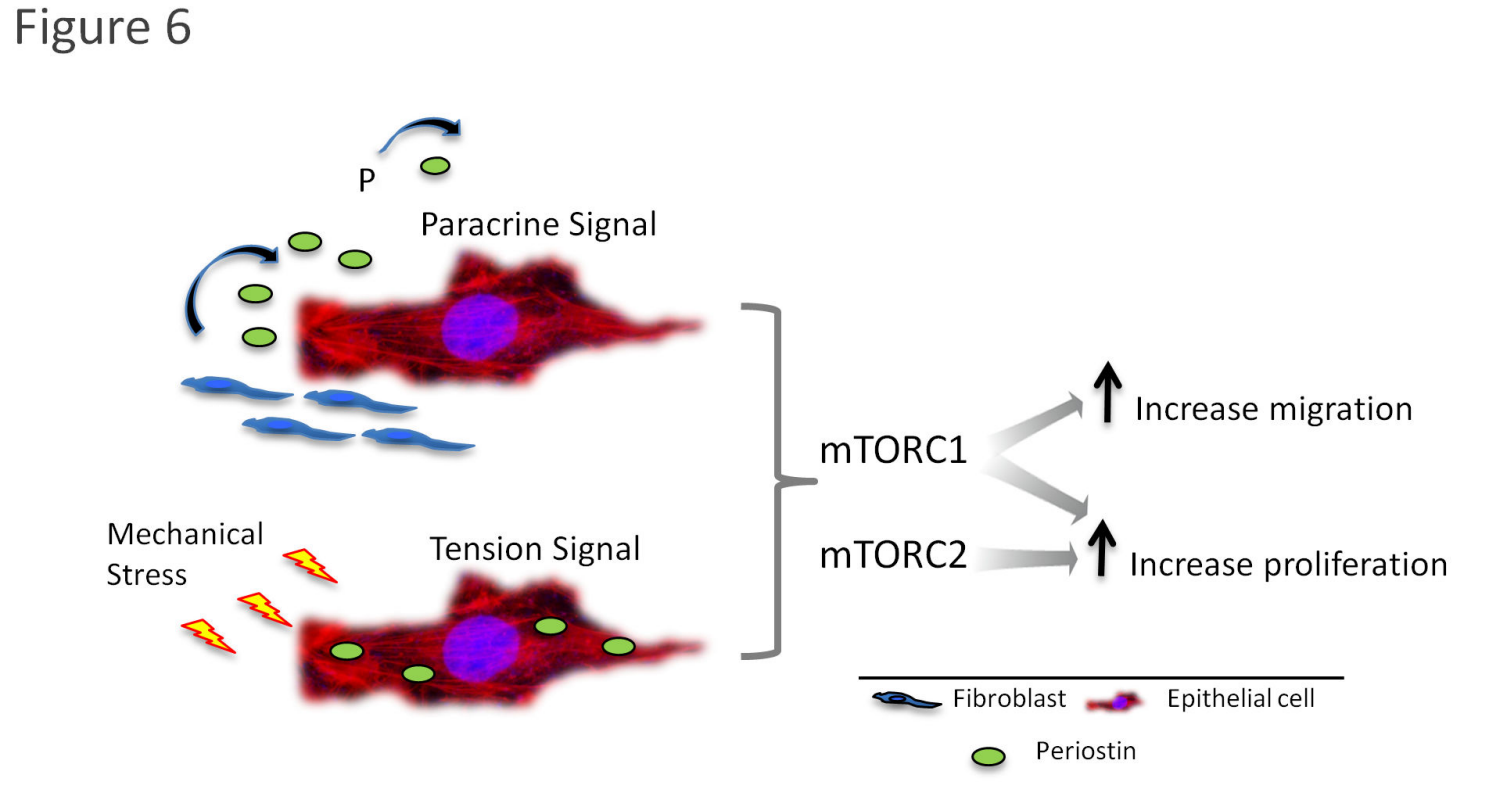
Proposed mechanism of Periostin-mediated accelerated epithelial healing through the mTOR pathway. During wound healing, activation of the Periostin signaling circuitry is initiated by the extracellular accumulation of Periostin secreted by fibroblasts, and by intracellular periostin originated after mechanical stress. Following, activation of the mTOR pathway occurs. Notably, the mTORC1 is required for Periostin-driven accelerated epithelial migration, while activation of mTORC1 and mTORC2 is required for epithelial proliferation.

Collectively, our results indicate that Periostin responds to mechanical stress generated during the wound healing process. Following Periostin production and secretion by fibroblasts, it generates a paracrine effect on keratinocytes. Keratinocytes respond to Periostin stimuli by migrating via mTORC1-dependent regulation, and by proliferating via mTORC1- and mTORC2-dependent mechanisms (Figure 6). 

## Discussion

The process of wound healing is characterized by early proliferation and migration of keratinocytes that play a fundamental role in a well-orchestrated mechanism that involves continuous exchange of signaling with the underlying stroma. Skin healing is primarily supported by the interaction of epithelial cells with the newly deposited extracellular matrix that is rich in secreted factors that are released in the wound bed. Many of these factors are associated with key cellular functions, such as migration, proliferation, directional migration, and cellular response to local mechanical stress, as shown here. Indeed, we found that expression of Periostin induced accelerated epithelial migration and proliferation through a process that involves polarization of actin filaments and activation of the mTOR signaling pathway. 

The matricellular protein Periostin is normally expressed in adult skin and is highly upregulated during epidermal healing [6,8,9,13,29]. In this study, we showed that Periostin expression was associated with the activation of epithelial stress markers and was found in the proliferative epithelial tongue that is localized in the wound. Both the CK6 stress marker and Periostin expression were confined to the proliferative and migratory component of the epithelial tongue, as determined by increased BrDU positive cells. Interestingly, the localization and expression pattern of Periostin were dynamically altered according to their proximity to the wound edge. Although Periostin was exclusively expressed at the basal membrane under the skin, actively migrating epithelial cells show remarkable cytoplasmic expression of Periostin, suggesting distinct roles dependent on localization. Therefore, the mitogenic and migratory capacity of epithelial cells during wound healing may not be solely driven by cell intrinsic signaling events, but may be the result of cooperative signaling pathways derived from epithelial and stromal skin components. Indeed, *in vitro* characterization of the process revealed that epithelial cells did not secrete Periostin, but they produced and accumulated low amounts of full-length Periostin. In contrast, fibroblasts produced and secreted large amounts of a variant isoform of Periostin. Indeed, Periostin is primarily associated with fibroblast rich tissues [47], suggesting that its splice variants are preferentially secreted and transduce signals to different tissues and cell types (reviewed in 16). We found that epithelial cells responded to exogenous Periostin by activating cellular proliferation and migration in combination with polarization of actin filaments. These data corroborate to the theory that epithelial migration during wound healing is not only controlled by intrinsic factors, but also by extrinsic stimuli compartmentalized in the wounded site, that promotes the re-epithelialization process [48]. Indeed, fibroblasts exposed to environmental changes during wound healing differentiate into α-SMA myofibroblasts, which enhance Periostin secretion [6,8,13].

 As a component of the extracellular matrix, Periostin interacts with integrin molecules that activate the PI3K signaling pathway in tumor cells [49,50]. Recent advances in the field of wound healing, driven by the use of genetically defined animal models capable of dissecting individual components of the PI3K pathway, have unveiled the molecular framework underlying skin homeostasis and tissue regeneration [23,24]. mTOR signaling is an integral component of the normal process of cutaneous healing and inhibition of mTOR results in delayed healing [23,51-53]. In this study, we exploited the ability of Periostin to activate the mTOR pathway, which is involved in a multitude of cellular functions, including cell growth, proliferation, motility, protein synthesis and transcription [54]. Surprisingly, we found that Periostin is colocalized with the mTOR readout marker pS6 at the leading edge of unstimulated epithelial cells. 

Periostin is overexpressed in different anatomical locations in the human body that are typically under mechanical stress, such as the periodontal ligament and heart valves [10,11]. Artificial mechanical stimulation of epithelial cells mediated by a computer-regulated bioreactor that applies “*in vitro*” cyclic tensile strains resulted in the simultaneous expression of Periostin and pS6, demonstrating a novel regulatory mechanism of Periostin and mTOR signaling in epithelial cells under mechanical stress. 

The clinical implications of these findings range from a deep understanding of the mechanism of Periostin in physiological conditions to the potential pharmacological intervention of Periostin in diseases and conditions like congenital ocular pathologies, persistent fetal vasculature [55], idiopathic pulmonary fibrosis [56], periodontal disease [57], and ventricular rupture after myocardial infarction [58] among others. Nonetheless, rapamycin and other FDA approved rapalogs constitute a viable therapeutic strategy for diseases and pathologies associated with increased levels of Periostin, including colorectal, prostate, breast, and head and neck cancers [59-62]. 
